# Preoperative angiographic considerations and neurological outcome after surgical treatment of intradural spinal hemangioblastoma: a multicenter retrospective case series

**DOI:** 10.1007/s11060-022-04213-2

**Published:** 2022-12-25

**Authors:** Vicki M. Butenschoen, Maximilian Schwendner, Vanessa Hubertus, Julia Onken, Nikolaus Koegl, Theresa Mohme, Stefanie Maurer, Tobias Boeckh-Behrens, Sven O. Eicker, Claudius Thomé, Peter Vajkoczy, Marcus Czabanka, Bernhard Meyer, Maria Wostrack

**Affiliations:** 1 Department of Neurosurgery, School of Medicine, Technical University Munich, Klinikum Rechts der Isar, Ismaningerstr. 22, 81675 Munich, Germany; 2 Department of Neurosurgery, Charité-Universitätsmedizin Berlin, Corporate Member of Freie Universität Berlin, Humboldt-Universität zu Berlin, Augustenburger Platz 1, 13353 Berlin, Germany; 3 Berlin Institute of Health, Augustenburger Platz 1, 13353 Berlin, Germany; 4 Department of Neurosurgery, Medical University of Innsbruck, Anichstr. 35, 6020 Innsbruck, Austria; 5 Department of Neurosurgery, University Medical Center Hamburg Eppendorf, Martinistraße 52, 20251 Hamburg, Germany; 6 Department of Neurosurgery, University Hospital Frankfurt, Theodor-Stern-Kai 7, 60590 Frankfurt am Main, Germany; 7 Department of Neuroradiology, School of Medicine, Technical University Munich, Ismaningerstr. 22, 81675 Munich, Germany

**Keywords:** Spinal hemangioblastoma, Vascularization, Embolization, Neurological outcome

## Abstract

**Purpose:**

Intradural spinal hemangioblastomas are rare highly hypervascularized benign neoplasms. Surgical resection remains the treatment of choice, with a significant risk of postoperative neurological deterioration. Due to the tumor infrequency, scientific evidence is scarce and limited to case reports and small case series.

**Methods:**

We performed a retrospective multicenter study including five high-volume neurosurgical centers analyzing patients surgically treated for spinal hemangioblastomas between 2006 and 2021. We assessed clinical status, surgical data, preoperative angiograms, and embolization when available. Follow-up records were analyzed, and logistic regression performed to assess possible risk factors for neurological deterioration.

**Results:**

We included 60 patients in Germany and Austria. Preoperative angiography was performed in 30% of the cases; 10% of the patients underwent preoperative embolization. Posterior tumor location and presence of a syrinx favored gross total tumor resection (93.8% vs. 83.3% and 97.1% vs. 84%). Preoperative embolization was not associated with postoperative worsening. The clinical outcome revealed a transient postoperative neurological deterioration in 38.3%, depending on symptom duration and preoperative modified McCormick grading, but patients recovered in most cases until follow-up.

**Conclusion:**

Spinal hemangioblastoma patients significantly benefit from early surgical treatment with only transient postoperative deterioration and complete recovery until follow-up. The performance of preoperative angiograms remains subject to center disparities.

## Introduction

Spinal hemangioblastomas are rare tumors and constitute only 2–6% of all primary spinal cord tumors [1–3]. They may appear sporadically or may be associated with a familiar neoplastic condition with autosomal dominance, the *von Hippel-Lindau* syndrome (VHL) [4–6], exhibiting other tumorous lesions such as cerebellar and retinal hemangioblastoma, abdominal pheochromocytoma, renal cell carcinoma, pancreatic cysts, and neuroendocrine tumors. [7] Hemangioblastomas present highly vascularized benign lesions and syringomyelia is often present. [8, 9] The treatment of choice remains the complete surgical resection of the tumor [10] in symptomatic patients suffering from VHL, and as early as possible in patients without VHL for diagnostic purposes and curative treatment [8]. Although the value of preoperative embolization has been demonstrated in cerebellar hemangioblastoma [11, 12], data on spinal intradural hemangioblastoma embolization remains sparse and limited to case reports. [13, 14] Up until now, no recommendation has been established regarding the preoperative embolization of spinal hemangioblastomas, and its use is limited to individual interdisciplinary discussions. We herein provide a large multicentered study analyzing the vascularization and operative outcome of spinal hemangioblastoma patients, focusing specifically on potential benefits of preoperative angiography and embolization.

## Methods

### Study cohort

We conducted a retrospective analysis of all consecutive patients treated surgically for histologically proven spinal hemangioblastoma World Health Organization Classification of the Central Nervous System WHO CNS ° 1 between January 2001 and July 2021. Patients were only included if complete preoperative and postoperative data were available.

### Study design

We performed a retrospective multicenter analysis including five neurosurgical high-volume centers in Germany (four University Hospitals: Munich, Berlin, Hamburg, Frankfurt) and Austria (University Hospital of Innsbruck). We assessed relevant details on the neurosurgical approach, the preoperative clinical status (modified McCormick scale [mMS], postoperative and long-term neurological outcome. Furthermore, we analyzed tumor size and localization on preoperative imaging (intramedullary, intra- and extramedullary, only extramedullary, anterior vs. posterior tumors), syringomyelia (T2 hyperintense cavity adjacent to the tumor lesion) and other tumor manifestations in cases of VHL patients, the extent of resection (EOR: gross total resection [GTR], subtotal resection [STR]), and time to tumor recurrence. We assessed preoperative digital subtraction angiography (DSA) if available, we identified specific vascular supply, and preoperative embolization, and its influence on postoperative outcome and complications.

### Statistics

For statistical analyses, we used SPSS Statistics 28 (IBM, Chicago, IL). Categorical data were compared using the chi-square test or Fisher’s exact test. Mean values were compared using the independent samples *t* test. We analyzed the association between potential factors and transient or permanent postoperative impairments (follow-up data or discharge data for those with missing follow-ups) using ANOVA and linear regression modeling. In addition, we assumed the following factors to be potentially predictive: tumor localization, anterior versus posterior tumor manifestation, VHL, EOR, preoperative embolization and age for univariate and multivariate analysis. Correlations were assessed with Kendall’s tau correlation coefficient. All tests were performed two sided at the 5% significance level.

### Ethical considerations

The presented study was performed in accordance with the ethical standards outlined in the Declaration of Helsinki. A local ethics committee gave a positive voice beforehand (number 5766/13). Due to the retrospective nature of the study, prospective patient consent was not required, and the local ethics committee (Prof. Georg Schmidt) waived it.

## Results

### Patient population

In total, we included 60 patients undergoing 69 operations over a period of 20 years for statistical analysis. Median age was 51 years (range 23 to 76 years), 55% patients were male (Table 1). Overall, 16 patients suffered from genetically confirmed VHL syndrome (26.7%); in 63.3%, VHL was excluded and in 10% the VHL status was unknown (six patients). Associated tumor manifestations included: cerebellar hemangioblastoma in 14/16 patients, retinal capillary hemangioblastomas in 4/16 patients, abdominal lesions in 5/16 patients including pancreatic and renal cysts as well as renal cell carcinoma.

**Table 1:** Demographics of our cohort population

	%	Range
Female sex (%)	45	
Age (median)	51	23–76
Level (%)		
Cervical	41.7	
CTJ	15	
Thoracic	18.3	
TLJ	6.7	
Lumbar	10.0	
Lumbosacral	3.3	
Ubiquitous	5.0	
Number of segments (median)	2	1–10
VHL (% confirmed)	26.7	
Localization		
Intramedullary	48.3	
Intra/extramedullary	36.7	
Extramedullary	15	
PreOP DSA (%)	30	
PreOP embolization (%)	10	
Syrinx (%)	56.7	

*CTJ* cervicothoracic junction, *TLJ* thoracolumbar junction, *VHL* von Hippel Lindau Syndrome, *DSA* digital subtraction angiography

Seven patients underwent multiple surgeries for spinal hemangioblastoma (11.7%, all VHL). In almost half of the patients, the tumor was localized strictly intramedullary (n = 29, 48.3%); intra- and extramedullary tumors were found in 36.7%; and strictly extramedullary tumors in 15% of the patients (n = 9). Median symptom duration was 24 weeks, depending on tumor location (anterior tumor location: 10 weeks; posterior tumor location: 41 weeks; intramedullary lesions: 27 weeks; and extramedullary tumor location: 12 weeks, p = 0.086).

Most patients suffered from tumors located at the cervical spine (41.7%), followed by the thoracic spine (18.3%), the cervicothoracic junction (15%), and the lumbar spine (10%) (Table 1). Median symptom duration was 6 months (interquartile range (IQ) 8 to 104 weeks).

A syrinx within the spinal cord was detected in 57.6% of the cases (34/59 patients, one patient did not undergo a preoperative MRI).

### Surgical approach

Tumor resection was achieved by a unilateral approach in 21 cases (35%), laminectomy in 14 cases (23.3%), and laminoplasty in 23 cases (38.3%) on a median length of two spinal levels (range 1–10 segments). Two patients (3.3%) required a dorsal instrumentation due to the tumor’s invasiveness and localization.

All patients underwent intraoperative neuromonitoring during tumor resection. Mean surgery duration was 197 min (range 82 to 612 min) and highly depended on the number of segments (p < 0.001). GTR and STR were accomplished in all patients (GTR 91.7% and STR 8.3%). No patient underwent a biopsy. Overall, five patients underwent a surgical revision for postoperative hematoma evacuation (two patients), cerebrospinal fluid leakage (two patients), and deep wound infection in one patient, resulting in an 8.3% surgical revision rate.

### Vascular supply

Preoperative DSA were available in 30% of the patients (18 patients), while preoperative embolization was only performed in 6/18 patients (10% of all patients). Reasons for non-embolization included tortuous vascularization without possibility for therapeutic intervention, radiculopial vessels, or non-identification of specific arterial feeders and unacceptable high risk of spinal cord ischemia. Preoperative embolization was performed in six patients, including the right vertebral artery (cervical hemangioblastoma), segmental arteries (thoracic, two patients; lumbar, one patient), and internal iliac as well as sacral arteries (two patients). Half of the patients undergoing a preoperative embolization suffered from lumbar or lumbosacral tumors.

Patients undergoing a preoperative embolization had larger tumor lesions (one segment: 4%; two segments: 11.5%; and three segments or more: 22%, p = 0.124), and exhibited a tumor extension including the anterior and posterior spinal cord (15.4%) and extramedullary tumors (33.3%, p = 0.042), showing an association between tumor extension and performance of embolization for preoperative risk reduction.

### Outcome

#### Clinical outcome

Before surgery, most patients presented with only mild neurological symptoms (mMS Grade I and II in 46.7% and 36.7% of the cases, respectively). Moderate impairment was detected in eight cases (mMS Grade III in 13.3%) and severe deficits in two patients (mMS Grade IV and V in 3.3%). Around 30% of the patients reported on preoperative motor deficits, while more than half of the patients complained of sensory deficits (56.7%). Gait disturbances were identified in 43.4%, and sphincter dysfunction in 13.3% of the population. After surgery, 78.3% of the patients remained clinically independent (mMS Grade I and II); the number of patients suffering from severe impairment increased from two to five patients (8.3%, mMS Grade IV). We observed a postoperative neurological deterioration in 23 patients (38.3%) with new gait disturbances in 11.6% of the patients, accentuated or new sensory deficits in 31.7% and new motor deficits in 13.3% of the cases.

**Fig. 1:**
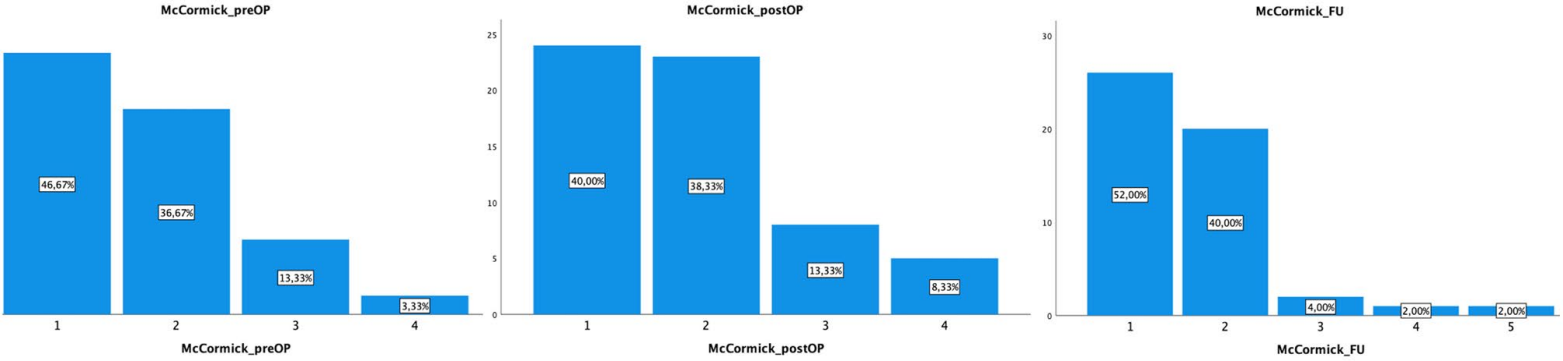
Modified McCormick (mMS) Grade pre-, postoperatively and at a median follow-up of 14 months, showing a postoperative deterioration with rapid clinical recovery and clinically independent patients in 92% of the cases

A clinical and radiological follow-up was available in 51/60 patients after a median time of 14 months (IQ range 4–42 months). Most patients recovered from their postoperative deterioration; 90.2% of the cases were declared mMS Grade I (52%) or II (40%) (Fig. 1). Only three patients had severe deficits (Grade IV, two patients) or were paraplegic (Grade V, one patient suffering from VHL with local and ubiquitous tumor progression and the interdisciplinary decision for best supportive care). Only 25.5% of the patients continued to suffer from motor deficits, while sensory deficits remained in 60.8% of the cases. Gait disturbances were described in 37.3% of the patients, and the clinical outcome overall improved in 39.2%. This was stable compared to the preoperative situation in 45.1% of the patients, and worsened in 15.7% (eight patients, including five patients suffering from tumor recurrence p = 0.003) (Table 2). At follow-up, 7.8% of the patients had suffered a tumor recurrence (depending on the EOR, GTR 4.3% vs. STR 50%, p = 0.001).

**Table 2:** Prognostic risk factors for postoperative neurological deterioration with their corresponding p-value

	Neurological Deterioration after surgery		p
	Yes	No	
**Symptom duration** **Mean** **(Median in weeks)**	197 (52)	69 (12)	0.031*
**Female Sex**	29.6	70.4	0.287
**Age (yrs) mean** **Age (yrs) median**	49 50	51 51	0.654
**Nr of segments (median)**	2	2	0.969
**Multiple spinal lesions**	40%	60%	0.537
Preoperative McCormick			
I	25%	75%	0.039*
II	45.5%	54.5%	
III	75%	25%	

#### Prognostic factors

Preoperative embolization and VHL did not significantly influence the occurrence of neurological deterioration after surgery (50% vs. 37%, p = 0.536; and 31.3% vs. 42.1%, p = 0.729). Patients suffering from extramedullary hemangioblastomas were less at risk for a neurological deterioration (11.1%) compared to patients suffering from intramedullary tumors (43.1%, p = 0.049). Neurological deterioration was significantly associated with the preoperative mMS Grade, with a higher percentage of patients deteriorating in the moderately impaired patient cohort (mMS Grade I: 25% deterioration, mMS Grade II 45.5% and mMS Grade III 75% deterioration, p = 0.039) and patient suffering from a longer symptom duration being at a higher risk (median 12 vs. 52 weeks, p = 0.031). Age did not increase the risk for a postoperative neurological deterioration, and the prevalence of multiple spinal lesions at the initial MRI did not increase the risk (Table 2). The number of segments was significantly associated with the prevalence of preoperative motor deficits (one segment: 12%, two segments 34.6% motor deficits, three segments or more: 66.7%, p = 0.007), but not with the postoperative deterioration, presumably due to preexisting deficits before surgery.

In univariate analysis, we found a significant association between the surgical approach and postoperative neurological deterioration (neurological deterioration was 60.9% in patients undergoing laminoplasty vs. 28.6% in patients undergoing laminectomy, p = 0.035). Yet, this association was only present in univariate analysis. In multivariate analysis, we detected a significant association between tumor localization and the surgical approach (only intramedullary tumors: 48.3% laminoplasty approach vs. 0% in extramedullary tumors, p = 0.003; and Roy’s Largest Root multivariate testing p = 0.033, partial eta square association 0.156).

GTR was achieved in 91.7% of the patients (55/60) and STR in 8.3%. The EOR was significantly associated with the tumor size (number of segments: one segment 100% GTR, two segments 88.5% GTR, three segments or more 77.8% GTR, p = 0.031), and dependent on tumor location (anterior intramedullary location 83.3% GTR, posterior location 93.8%). The presence of a syrinx favored GTR (97.1% vs. 84%), but the association failed to reach statistical significance (p = 0.075).

The mMS grades at follow-up was significantly dependent on the EOR: patients undergoing GTR were only mildly impaired in 93.7% (mMS Grade I or II) while only 50% of the patients undergoing STR were clinically independent (mMS Grade I or II), possibly due to tumor recurrence (p < 0.001).

#### Case presentation

**Fig. 2:**
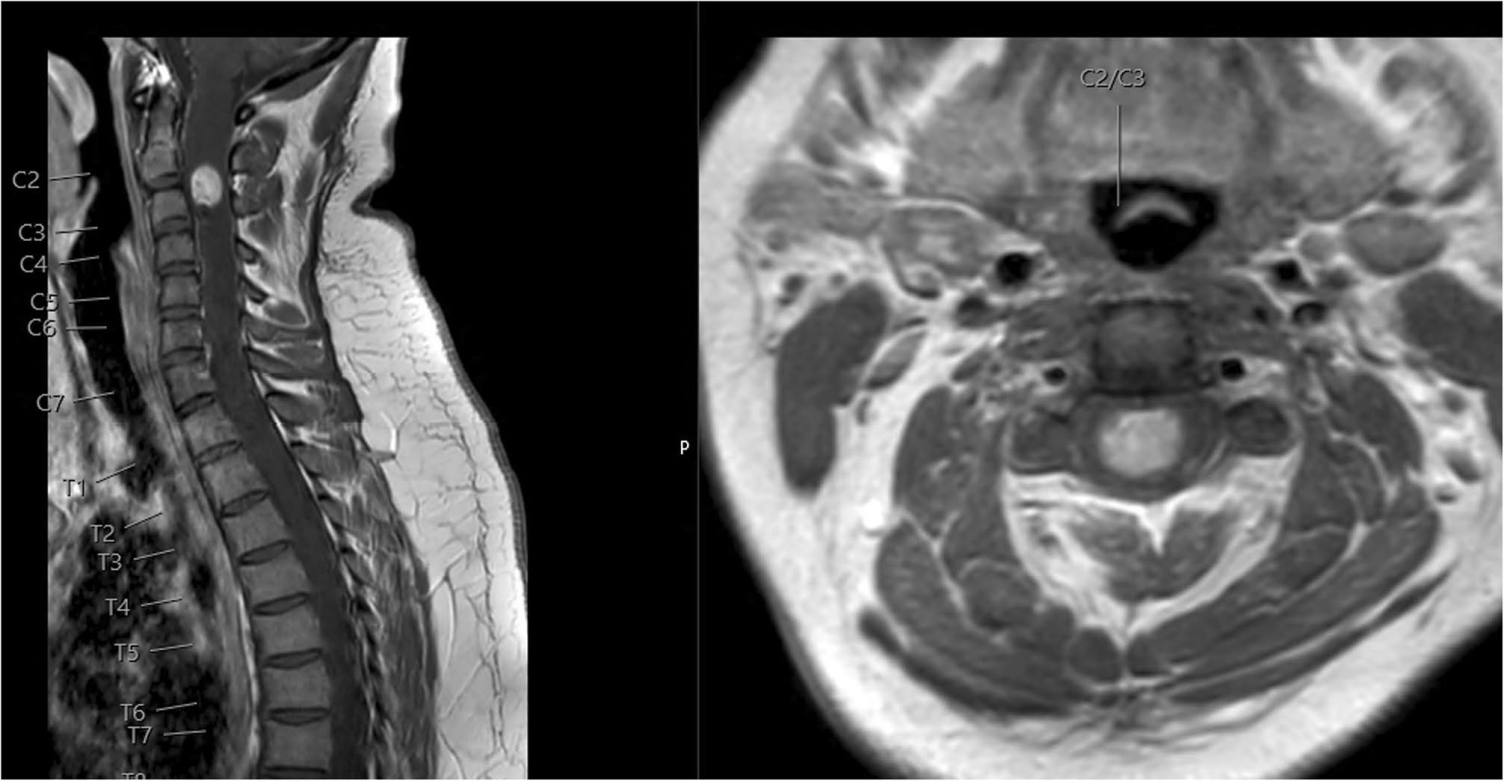
T1-MRI scan with contrast enhancement showing a contrast-enhancing lesion reaching from C2 to C3 with extensive vascularization

**Fig. 3:**
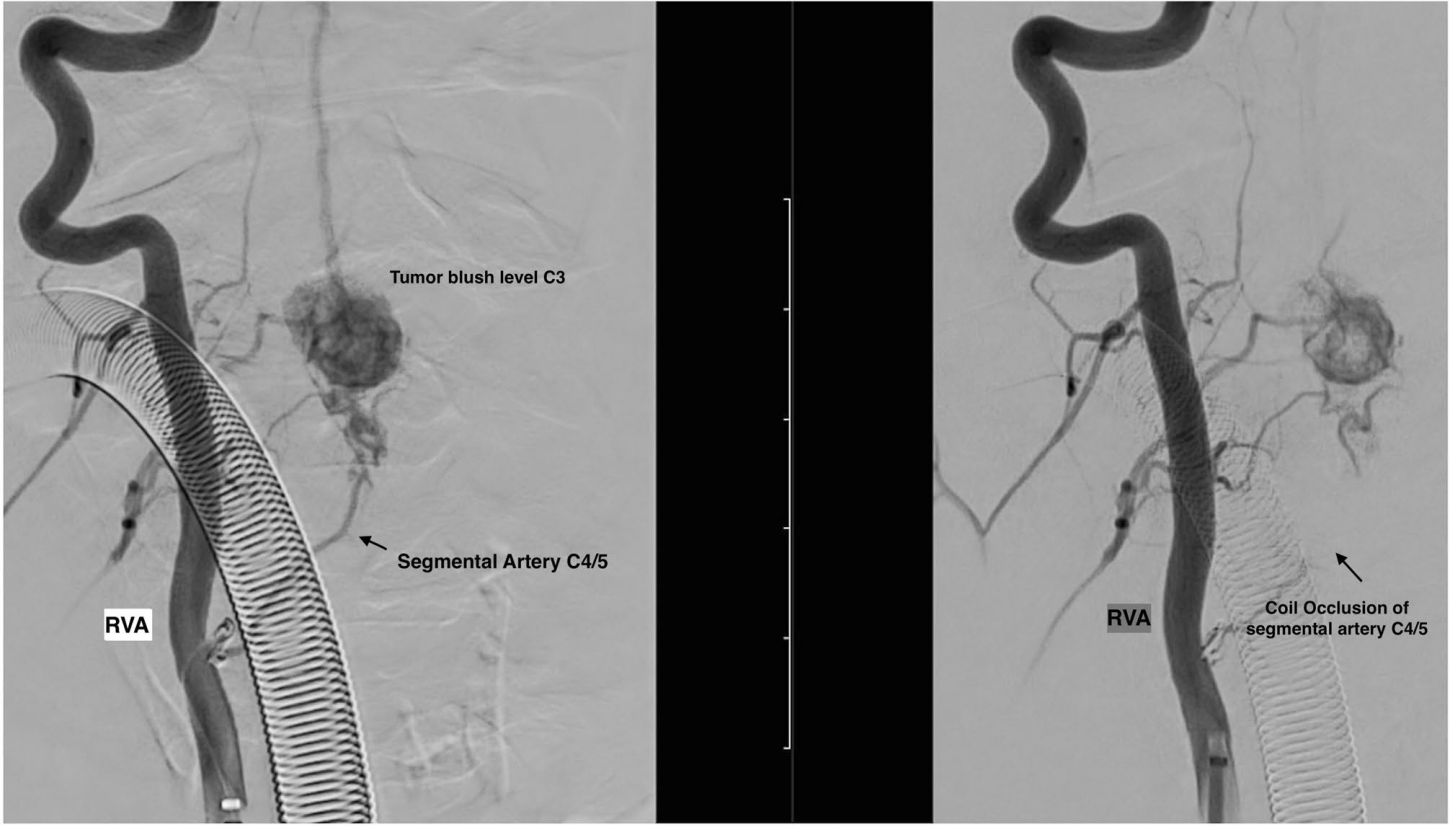
Preoperative angiography showing successful partly embolization (left image before embolization, right image after embolization of the feeding vessel)

**Fig. 4:**
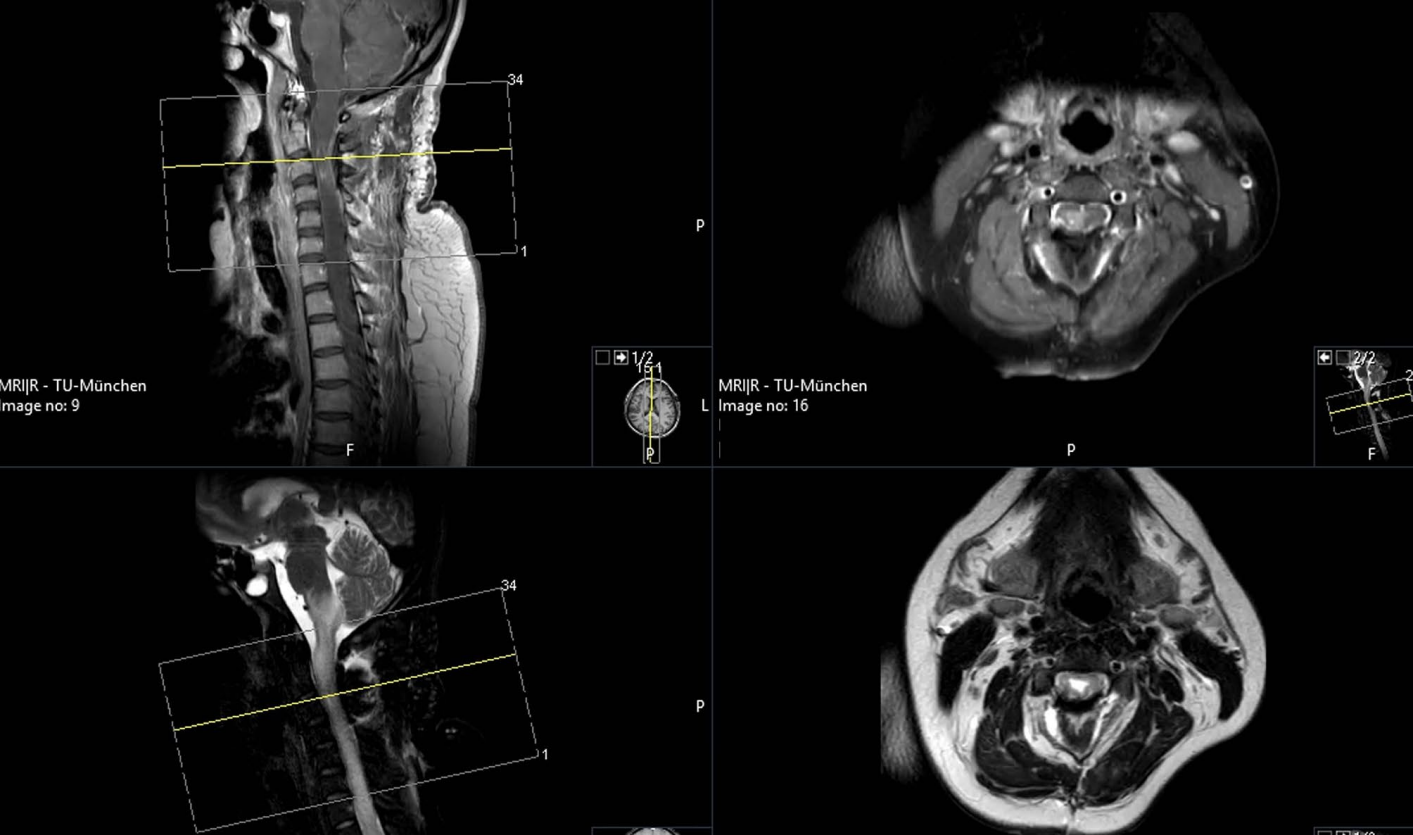
T1-MRI scan with contrast enhancement (upper image) and T2-MRI scan (lower image) confirming complete resection of the lesion

A young female patient initially presented with progressive neck pain and recurrent dysesthesia of both hands. She showed no neurological deficits. A contrast enhanced MRI revealed an intramedullary lesion at the cervical level reaching from C2-C3 raising the suspicion of a spinal hemangioblastoma (Fig. 2). A preoperative DSA showed an increased vascularization of the lesion, and the patient underwent preoperative embolization of the tumor feeding segmental artery (Fig. 3). Surgical complete tumor resection was performed via laminoplasties C2-3 under IONM (Fig. 4). After surgery, we observed a transient worsening of the patient’s motor function (right sided hemiparesis MRC 3/5. At follow-up, the patient recovered from her postoperative neurological deterioration (MRC5/5) and remains recurrence free two years after surgery.

## Discussion

### Clinical and surgical outcome

Neurological deterioration after surgical resection of spinal hemangioblastomas occurred in as many as 38.3% of the patients. Although neurological deterioration is believed to be transient and usually resolves by the time of follow-up [1], the high percentage is congruent with the published literature, reporting up to 40% experienced deterioration of preoperative neurological status in the immediate postoperative period. [1] Similar to the published literature, the postoperative impairment was only transient, and patients recovered until follow-up.

Congruent with the published literature, patients presenting with an associated syrinx had a favored EOR, probably due to a displacing rather than an infiltrating tumor [9]. In our patient cohort, the share of GTR was higher in patients presenting with a syrinx on the preoperative MRI compared to patients without radiological signs of a syrinx, but this was probably due to the small patient cohort; the difference failed to reach statistical significance.

Previous publications described an association between the tumor’s location in relation to the spinal canal, differentiating between anterior (ventral) and posterior (dorsal) tumor manifestations. Previous studies showed a potential relationship between ventral tumors and the increased risk of postoperative worsening (with an Odds Ratio of up to 15.66 [15]). In our cohort population, we did not identify the same association regarding tumor location and postoperative worsening (p = 0.740). Considering we did identify a center effect here; the lack of statistical difference may be biased.

Published literature has mentioned an association between the surgical approach and the clinical outcome [16], favoring laminotomies over laminectomies in univariate analysis. In our patient cohort, we also identified a significant association between the surgical approach and the postoperative clinical outcome. In multivariate analysis, the association was not significant. We therefore question its clinical and statistical value. In multivariate testing including anterior versus posterior location and intra- versus extramedullary tumors, the association was not statistically significant, highlighting the importance of multivariate testing regarding postoperative deterioration.

Considering the preoperative mMS grades and duration of symptoms significantly affected the risk for a postoperative neurological deterioration, we strongly advise asymptomatic patients without VHL to obtain early surgical treatment, which is a recommendation the published literature supports. [8, 17]

### Vascularization

In our cohort, only six patients underwent a preoperative embolization, half of them at the lumbar or lumbosacral level where the risk of a spinal cord ischemia or spinal cord edema and swelling was presumably low. The rate of preoperative DSA was clearly associated with tumor size, indicating a tendency to perform a preoperative DSA in larger tumors with a higher risk of intraoperative bleeding due to rich blood supply. Unfortunately, no scientific comparison can be made regarding the benefits of preoperative embolization and blood loss because we only analyzed six patients undergoing preoperative embolization in larger, mostly extramedullary tumor lesions.

### Limitations

Despite its multicenter nature, our study population was limited due to the rarity of this benign disease. The case number may be responsible for the lack of statistical significance in prognostic factors showing a trend without reaching a p-value less than 0.05 (such as the presence of a syrinx, GTR, and tumor location).

Due to the low rate of cervical or thoracic tumor embolization, our cohort may underestimate the risks of extensive preoperative embolization and its risk for postinterventional neurological deterioration. Therefore, prospective patient enrolment should be performed with a specific focus on preoperative embolization in spinal hemangioblastoma patients.

Considering we performed a multicentered study, center effects must be considered. We found no significant differences in spinal levels involved (p = 0.969), postoperative neurological deterioration (p = 0.162) and surgical revision rates (p = 0.249) depending on the center involved. Significant center differences were found for the performance of preoperative DSA (p = 0.037) and tumor localization (p = 0.020), showing disparities regarding preoperative imaging, the availability of preoperative embolization and anterior versus posterior tumor localizations.

In our retrospective cohort, we did not compare different surgical treatment options because all patients aimed for a GTR; we also did not analyze patients undergoing radiotherapy [18] because radiotherapy is usually not recommended as a first-line treatment in our departments. Current literature supports our treatment algorithm, as radiotherapy should be reserved for treating tumors that are not surgically resectable [19]. Neither did we investigate the role of IONM, nor the role of Indocyanine Green Angiography during surgery [20].

## Conclusion

Patients suffering from spinal hemangioblastomas may experience a transient postoperative neurological deterioration in more than one third of the cases. Preoperative clinical status and symptom duration present important risk factors, and early surgical treatment should be offered to patients without VHL. Postoperative neurological deterioration remains transient in most cases. Although evidence is sparse, patients with large tumor manifestations may benefit from preoperative embolization and preoperative DSA should be considered accordingly.

## Data Availability

The data that support the findings of this study are available on request from the corresponding author.
